# PPAR**α** as a Transcriptional Regulator for Detoxification of Plant Diet-Derived Unfavorable Compounds

**DOI:** 10.1155/2012/814945

**Published:** 2012-04-19

**Authors:** Bunichiro Ashibe, Yu Nakajima, Yuka Fukui, Kiyoto Motojima

**Affiliations:** ^1^Department of Biochemistry, Meiji Pharmaceutical University, 2-522-1 Kiyose, Tokyo 204-8588, Japan; ^2^Central Research Center, Yomeishu Seizo Co., Ltd., Minowa, Nagano 399-4601, Japan

## Abstract

Plants contain potentially toxic compounds for animals and animals have developed physiological strategies to detoxify the ingested toxins during evolution. Feeding mice with various plant seeds and grains showed unexpected result that only sesame killed PPAR**α**-null mice but not wild-type mice at all. A detailed analysis of this observation revealed that PPAR**α** is involved in the metabolism of toxic compounds from plants as well as endobiotic substrates by inducing phase I and phase II detoxification enzymes. PPAR**α** plays a vital role in direct or indirect activation of the relevant genes via the complex network among other xenobiotic nuclear receptors. Thus, PPAR**α** plays its wider and more extensive role in energy metabolism from natural food intake to fat storage than previously thought.

## 1. Introduction

Animals feed on plants for nutritional and tasting reasons. Plants and seeds are important energy sources but they also contain unique compounds and a variety of secondary metabolites some of which are potentially toxic to animals [1]. Chlorophyll, for example, is a green pigment essential for conversion of the energy of sunlight into chemical energy but its derivative contains a branched fatty acid, phytanic acid, which is toxic at least for rodents and human. Popular plant toxins include flavonoids, isoflavonoids, and tannins, but there should be much more. Animals have developed physiological strategies to avoid specific plants and to detoxify the ingested toxins. Physiological detoxification can occur in the mouth and the gut rumen with or without a help of microbes and once absorbed toxins must be detoxified in the intestine and liver [2]. Phytanic acid is completely broken down in the liver and a fatty aldehyde dehydrogenase, FALDH encoded by *ALDH3A2*, plays a key role in the process [3–5] as its defect causes Sjögren-Larsson syndrome [6, 7]. However, identification of potentially toxic compounds in plants and seeds is incomplete and the mechanisms of detoxification steps have not been well characterized yet because most animal studies to date have been carried out using artificial laboratory diets. Recently, we found that a nuclear receptor peroxisome proliferator-activated receptor *α* (PPAR*α*) plays a vital role in induction of a detoxification system by using natural plant seeds as diets for mice [8].

An important role of PPAR*α* in lipid catabolism in the liver has been well established [9], but the PPAR*α*-null mouse shows little phenotypic change when fed with normal laboratory diet [10, 11]. We searched its extrahepatic roles and found that PPAR*α* is essential to induce 17*β*-hydroxysteroid dehydrogenase type 11 (17*β*HSD11) in the intestine [12]. Studies on the substrate specificity of 17*β*HSD11 showed that it includes not only glucocorticoids and sex steroids but also bile acids, fatty acids, and branched amino acids [13, 14]. So we examined the possibility that PPAR*α* plays a vital role in inducing enzymes for metabolizing secondary metabolites of plants by feeding natural plant seeds. We begin this review by summarizing our results of experiments using various plant seeds as diets for wild-type mice and PPAR*α*-null mice. The main focus of this review is on the role of PPAR*α* in inducing a detoxification system against potentially toxic plant compounds.

## 2. All PPAR*α*-Null Mice Died within 40 Days of Sesame Diet

Sesame is widely thought to be a healthy food and it contains several lignans and lignan glycosides that have antioxidant properties [15, 16]. Thus, our result that feeding the PPAR*α*-null mice with sesame seeds for a short period sometimes caused the death [8] was totally unexpected for us. We first examined whether the lethal effect of feeding sesame to PPAR*α*-null mice was specific to sesame, or whether similar results could be caused by other plant seeds or nuts. For this study, almond (*Prunus dulcis*) nuts and sunflower (*Helianhus annuus*) seeds were chosen as control diets on the basis of their lipid contents. All are rich in lipids, but sesame seeds are rich in oleic acid and linoleic acid, whereas almond nuts are rich in oleic acid and sunflower seeds are rich in linoleic acid. Only sesame seeds among the three fatty seeds killed PPAR*α*-null mice in several days. This result suggests that a large amount of major fatty acids itself is not toxic for PPAR*α*-null mice but some sesame-specific or -enriched compounds may be involved in causing a lethal effect in the liver. However, major lignans and lignan glycosides in sesame seeds were not toxic.

Feeding these seeds and nuts induced the formation of lipid droplets with similar fatty acid compositions to those of the diets in the liver of both wild-type and PPAR*α*-null mice [17]. Thus, the fatty acid compositions of neutral lipids in hepatic lipid droplets were strongly influenced by those in the diet, suggesting that the lipid droplets promptly stored the diet-derived excess fats with unfavorable fatty acid compositions and protected the cells from lipotoxicity. No lethal effect of feeding almond nuts or sunflower seeds to PPAR*α*-null mice was observed. The fatty acid compositions of the hepatic lipids quickly returned to normal after changing the diet to the usual one in wild-type mice. This quick reversibility suggests that enhanced lipid metabolism to remodel the fatty acid composition occurred around the lipid droplets in the liver of wild-type mice. However, quick reversibility was not observed in PPAR*α*-null mice. These results suggest that PPAR*α* plays an important role in the rapid induction of lipid-metabolizing/remodeling enzymes according to the contents of the diet [17]. However, PPAR*α* may not be absolutely necessary for metabolism of major fatty acids but vital for detoxification of minor but sesame-enriched abnormal fatty acid(s) or compound(s).

**Figure 1:**
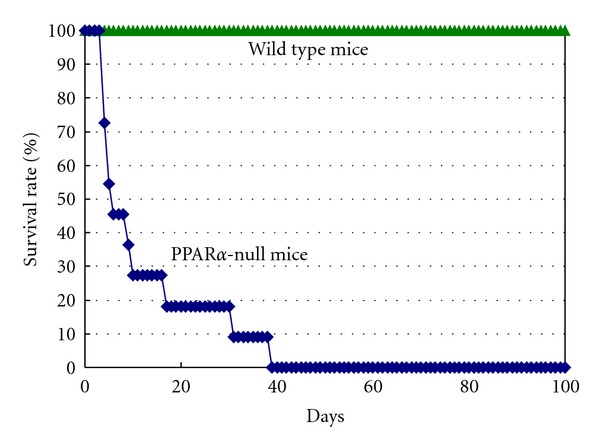
Survival curve of normal and PPAR*α*-null mice on sesame diet. Male PPAR*α*-null mice on the sesame diet were followed until all null mice (*n* = 12) died. None of wild-type mice (*n* = 4) on the sesame diet or the PPAR*α*-null mice on normal diet died during these period. Time 0 is the day of starting the experiment using age-matched (14 weeks) mice on the sesame diet.

We next examined the survival curve of mice on sesame diet for a long period. Feeding wild-type mice with sesame seed diet had no effect on survival for at least 3 months (not shown) but PPAR*α*-null mice started to die in a few days and all died within 40 days after starting the sesame diet (Figure 1). This drastic effect was age and gender dependent; only male PPAR*α*-null mice of older than 14 weeks of age died on the sesame diet. During these periods, weight gain was not observed in wild-type mice and a gradual weight loss was seen in PPAR*α*-null mice, indicating that living only on sesame seeds is not healthy even for wild-type mice.

The mechanism for the striking female-specific tolerance to the sesame diet can be similar to that reported by Djouadi et al. [18] for explanation of the result from a carnitine palmitoyltransferase1 (CPT1) inhibitor, etomoxir experiment using PPAR-null mice. Etomoxir treatment killed all male PPAR-null mice almost within 24 h, whereas no female PPAR-null mice nor wild-type mice died within the same period. They did not define the mechanism for the gender-specific response but they speculated that estradiol-triggered fatty acid utilization pathway is important in hepatic lipid and glucose homeostasis. Similar effects of estradiol and also of aging were reported by Duan et al. [19] in the expression of intestinal sterol transporters. Thus, the estradiol-dependent pathway may play an essential role in female-specific tolerance to the sesame diet.

## 3. The Sesame-Diet-Induced Severe Hypoglycemia in PPAR*α*-Null Mice

Feeding sesame seeds caused a rapid decrease in one day in the serum levels of glucose and in the glycogen contents in the liver of both mice although the reduction in PPAR*α*-null mice was much significant compared with wild-type mice [12]. After the reduction, wild-type mice recovered the serum glucose levels to normal, but the severe hypoglycemia persisted for several days in most PPAR*α*-null mice. Thus, the major cause of the sesame-induced death in PPAR*α*-null mice during a short period would be severe hypoglycemia. There seems to be no possibility that gluconeogenesis was blocked in the PPAR*α*-null mice on a sesame diet at the transcriptional level of the key enzymes in gluconeogenesis. Although feeding sesame seeds differently changed the levels of mRNAs for the key enzymes in gluconeogenesis (phosphoenolpyruvate carboxykinase (PEPCK), glucose-6-phosphatase (G6Pase), PPAR*γ*-coactivator 1*α* (PGC-1*α*), and aquaporins 3 and 9) between wild-type mice and PPAR*α*-null mice, these small changes could not fully explain the sesame-seed-induced persisting hypoglycemia in the PPAR*α*-null mice. Another possibility might be that ATP supply necessary for gluconeogenesis was not enough because mitochondrial function including *β*-oxidation of fatty acids in the liver was inhibited by some compounds from sesame seeds. The toxic compounds should have been metabolized in a PPAR*α*-dependent manner. In any case, acute hypoglycemia alone cannot explain chronic effect of feeding sesame seeds on PPAR*α*-null mice.

## 4. The Sesame-Diet-Induced FALDH in the Liver of Normal Mice but Far Less in PPAR*α*-Null Mice

**Figure 2:**
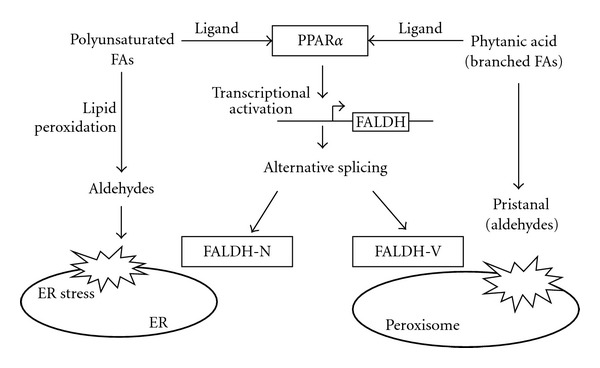
Proposed physiological role of FALDH. Polyunsaturated fatty acids or branched fatty acids bind to PPAR*α* to express FALDH and FALDH variants produced by alternative splicing share a role in protecting against oxidative stress in an organelle-specific manner.

Microarray analysis of PPAR*α*-dependent sesame-induced genes revealed that PPAR*α* plays a vital role in inducing various enzymes for protecting against oxidative stress and xenobiotic metabolism in the intestine and liver [8]. Fatty aldehyde dehydogenase (FALDH or ALDH3A2) was identified as one candidate enzyme induced in the liver for lipid peroxidation [20]. Results from our detailed analyses of the mechanisms for PPAR*α*-dependent transcriptional induction, subcellular localization [21], and protecting against lipotoxicity [22] are summarized in Figure 2. Both polyunsaturated fatty acids and branched fatty acids act as ligands for PPAR*α* and directly regulate expression of FALDH via a direct repeat-1 (DR-1) site located in the promoter region of the FALDH gene. Alternative splicing produces two isoforms, FALDH-N and FALDH-V. FALDH-N localizes on endoplasmic reticulum membrane and detoxifies polyunsaturated fatty-acid-derived fatty aldehydes and protects cells from endoplasmic reticulum stress. FALDH-V is a unique peroxisome-specific aldehyde dehydrogenase playing an essential role in oxidation of branched fatty acids including lipid peroxidation products and phytanic acid from plants.

The expression levels of major endoplasmic localizing FALDH-N and peroxisome-specific FALDH-V in wild-type and PPAR*α*-null mice fed normal or sesame-diet were examined by the real-time PCR analysis using the isoform-specific PCR primers. Both FALDH isoforms were induced several fold from the basal levels by feeding sesame regardless of whether PPAR*α* was expressed or not [21]. This result indicates that expression of FALDH is also regulated by a PPAR*α*-independent mechanism. However, the basal expression levels of both isoforms in PPAR*α*-null mice were quite low and even their induced levels were barely similar with the uninduced levels in wild-type mice. These results suggest that the FALDH activities in the liver of PPAR*α*-null mice were not high enough to protect from the oxidative stress caused by feeding the sesame diet.

## 5. The Sesame Diet Did Not Cause a Significant Oxidative Stress but Induced Hepatoxicity in PPAR*α*-Null Mice

We next directly examined the possibility that sesame-diet-induced oxidative stress in PPAR*α*-null mice by measuring the serum levels of 8-hydroxy-2′-deoxyguanosine (8OH-dG), an indicator of oxidative damage of DNA [23], and 8-isoprostane, an autooxidation product from arachidonic acid and a biomarker for oxidative stress [24]. The levels of 8OH-dG did not change significantly in wild-type or PPR*α*-null mice feeding normal or sesame diet. In contrast, the levels of 8-isoprostane in the sera of PPAR*α*-null mice were about 3-fold higher than those of wild-type mice even feeding normal diet (0.3–0.4 versus 0.4–1.4 ng/mL) and the sesame diet did not raise the levels significantly. These results indicate that the sesame diet does not induce a significant oxidative stress and that FALDH may not play an important role in protecting the cells from lipotoxicity induced by the sesame diet. However, they do not exclude a possibility that FALDH is important for metabolism of the sesame-derived toxic compounds.

17*β*HSD11 was also identified as a sesame-diet-inducible enzyme in a PPAR*α*-dependent manner [8]. 17*β*HSD11 is mostly localized on the endoplasmic reticulum membrane under normal conditions and redistributes to lipid droplets when their formation is induced by a lipid-rich diet [25, 26]. Its involvement in detoxification of lipophilic toxic compounds compartmentalized in lipid droplets is a plausible possibility. Thus, we think that FALDH and 17*β*HSD11 are important for detoxification of toxic compounds contained in sesame seeds rather than in protecting the cells from oxidative stress induced by a lipid-rich diet.

To assess liver damage after feeding sesame seeds, we measured the levels of alanine aminotransferase (ALT) and aspartate aminotransferase (AST) in the serum of wild-type and PPAR*α*-null mice [8]. Feeding the mice with sesame caused hepatotoxicity as indicated by measuring the plasma alanine transaminase (ALT) activities. At day 3 after starting the diet, ALT activities went up from 12.5 ± 5.0 (IU/L, ±S.D.) to 26.3 ± 2.5 in wild type mice and from 13.8 ± 4.8 to  253 ± 169  in PPAR*α* null mice, and AST also went up from  40 ± 7.5  (IU/L, ±S.D.) to  620 ± 220  in PPAR*α* null mice. These results suggest that expression of PPAR*α* protected the liver cells from the damages caused by feeding sesame seeds.

## 6. The Sesame-Diet-Induced Various Cyps in a PPAR*α*-Dependent and a PPAR*α*-Independent Manner

Microarray analysis was performed to identify the sesame-diet-induced detoxification enzymes in the intestine and liver by comparing the expression levels of mRNAs using RNA isolated from wild-type mice and PPAR*α* null mice fed with sesame for one week. Sesame seeds, like other botanicals [27–29], should contain a large number of compounds that affect cell function via gene transcription or metabolic inhibition. In addition to phase II detoxification enzymes such as UDP-glucuronosyltransferases (UGTs), aldoketoreductases (AKRs), glutathione S-transferases (GSTs), and drug transporters [8], the expression levels of several cytochrome P450 enzymes (Cyps) in the intestine and liver were much higher in wild-type mice than in PPAR*α*-null mice when they fed with sesame. These Cyps were induced in a PPAR*α*-dependent manner as confirmed by quantitative real-time PCR analysis although some of them have not been identified as PPAR*α*-target genes. Expressions of the corresponding human CYP2C9, 2B6, and 3A4 to these mouse Cyps were reported be regulated by the constitutive androstane receptor (CAR) [30, 31]. Jackson et al. [32] proposed an imperfect DR4 element as an essential element for CAR-dependent transcriptional activation of Cyp2c29 and 2b10 genes, although no detailed mechanism has yet been elucidated. Thus, the indirect but essential involvement of PPAR*α* in the induction of these Cyps can be at the activation step of CAR. Further analysis is clearly necessary to obtain direct evidence of the involvement of PPAR*α* in the activation step of CAR. Another possibility for the indirect but essential involvement of PPAR*α* in the induction is that some of them are regulated by overlapping transcriptional programs mediated by an axis of PPAR*α*- retinoid X receptor (RXR)and liver X receptor (LXR) as suggested by Anderson et al. [33]. Our observations of indirect but essential involvement of PPAR*α* in the transcriptional activation of several Cyp genes should provide an important clue to elucidate the activation processes and the complex network among the xenobiotic nuclear receptors [34–38].

Microarray analysis and quantitative real-time PCR analysis showed that most detoxification enzyme mRNAs were induced by the sesame diet in both the liver and intestine and the extents of induction were larger in wild-type mice than in PPAR*α*-null mice. However, Cyp2a5 was an exception because it was largely induced by the sesame diet in PPAR*α*-null mice but not all in wild-type mice (unpublished). It is plausible that certain compound(s) in sesame seeds triggered the transcriptional activation of these Cyp genes through an axis of nuclear receptors excluding PPAR*α*. The reason for the PPAR*α*-null mouse-specific induction of some Cyps can be explained by the mechanism that the relevant compounds should have been metabolized to inactive form(s) in the liver of wild-type mice in a PPAR*α*-dependent manner.

## 7. PPAR*α* as a Member of Transcriptional Regulators for Detoxification of Unfavorable Compounds

**Figure 3:**
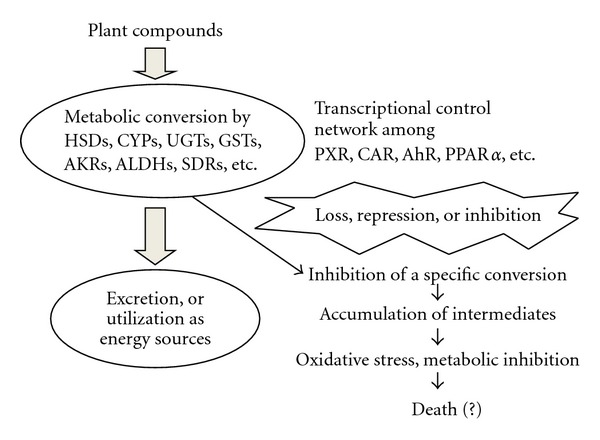
A proposed model depicting the metabolic conversion of plant compounds in animals by the mechanism involving complex network among the xenobiotic nuclear receptors and PPAR*α*.

In conclusion, the detoxifying enzymes in the liver must be induced by complex functional interactions among xenobiotic receptors, such as CAR, pregnelone X receptor (PXR), and aryl hydrocarbon receptor (AhR), to metabolize a large number of the plant compounds that affect cell function via gene transcription, or metabolic inhibition [8, 39, 40]. As summarized in Figure 3, PPAR*α* should be one of these nuclear receptor. One receptor may be involved in producing the metabolites/ligands for the next receptor that will be involved in inducing the enzymes for further metabolism. Disturbance of this network by genetic mutation, transcriptional repression or metabolic inhibition should severely affect metabolism of xenobiotics if it goes beyond compensating capacity coming from overlapping functions of metabolizing enzymes. PPAR*α* plays wider and more extensive role in energy metabolism form catabolism of endobiotic lipids but also for detoxification of xenobiotic or plant-derived unfavorable compounds than previously thought.
